# Draft Genome Sequence of Bacillus thuringiensis Strain m401, Isolated from Honey in Argentina

**DOI:** 10.1128/MRA.01158-18

**Published:** 2018-11-08

**Authors:** Eliana Abrahamovich, Mario E. E. Franco, Ana C. López, Adriana M. Alippi, Pedro A. Balatti

**Affiliations:** a Centro de Investigaciones de Fitopatología-CIDEFI (CICPBA), Facultad de Ciencias Agrarias y Forestales, Universidad Nacional de La Plata, La Plata, Buenos Aires, Argentina; Indiana University Bloomington

## Abstract

We report here the 6,092,003-bp draft genome sequence of Bacillus thuringiensis strain m401, a tetracycline-resistant isolate recovered from honey. The isolate contained three plasmids of 8,307 bp, 9,934 bp, and 69,561 bp and a tetracycline resistance gene with high homology to *tet45* in a contig of 236,180 bp.

## ANNOUNCEMENT

Spore-forming bacteria of *Bacillus*, and also Paenibacillus larvae, the causal agent of American foulbrood disease, are frequently present in honey (1, 2). This disease is controlled in beehives with oxytetracycline favoring the emergence of resistance within the bacterial population (3). Bacterial resistance to tetracyclines is mainly due to the presence of *tet* (tetracycline) and *otr* (oxytetracycline) genes frequently associated with mobile elements (4). Tetracycline-resistant strains of the genus *Bacillus* have been found in honey (5).

Bacillus thuringiensis strain m401 was recovered from honey samples from the province of Buenos Aires, Argentina (6). A 10-ml honey sample was homogenized at 40°C, diluted with phosphate buffer at pH 7.2, and centrifuged (3,500 × *g*; 45 min; 4°C). A 3-ml aliquot of the vortexed fluid-sediment mixture was heated to 80°C (10 min), and 10 µl was spread on Mueller-Hinton broth, yeast extract, potassium phosphate, glucose, and pyruvate (MYPGP) agar (6) and on Trypticase soy agar (TSA) (6) and incubated at 37°C and 30°C, from 48 h to 7 days. *Bacillus* species developed in the TSA plates.

Tetracycline resistance strain m401 has been used to study the mechanisms of tetracycline resistance transfer (7). Total DNA was obtained from overnight cultures grown on Mueller-Hinton broth at 32°C with aeration using the Wizard genomic DNA purification kit (Promega). The DNA solution was further purified using phenol/chloroform/isoamyl alcohol at a ratio of 25:24:1 (pH 8.0) and, subsequently, ethanol precipitation. The final pellet was resuspended in buffer (10 mM Tris-Cl, pH 8.5). The quality and quantity of DNA were assessed by gel electrophoresis using a NanoDrop 2000 UV-Vis spectrophotometer (Thermo Fisher Scientific, MA). A library was prepared using the TruSeq DNA library preparation kit, and 2 × 100-bp paired-end sequencing was performed on an Illumina HiSeq 4000 sequencing system at Macrogen Co. (Seoul, South Korea). Raw reads were error corrected and *de novo* assembled using SPAdes 3.11.1 (8). In addition, raw reads were quality trimmed using Trimmomatic 0.36 (9) and *de novo* assembled with Geneious 9.1.2 (10) using medium-low sensitivity, enabling the option to circularize contigs with matching. The quality of the genome assembly was assessed using QUAST (11). Open reading frames (ORFs), tRNAs, and rRNAs were predicted using Glimmer 3 (12), tRNAscan-SE 2.0 (13), and RNAmmer 1.2 (14), respectively. The functional annotation was carried out using Blast2GO Basic (15).

Sequencing yielded 1,987,020,672 bases in 19,673,472 reads. The genome was assembled into 45 scaffolds (>500 bp; *N*_50_, 391,966 bp) with an average coverage of 146×, comprising a total of 6,092,003 bp with a G+C content of 34.72%. The assembly included three circular contigs of 69,561 bp, 9,934 bp, and 8,307 bp. A total of 5,905 genes were annotated, including 3 rRNAs, 25 tRNAs, and 5,877 ORFs. InterPro accession numbers, Gene Ontology identification numbers, and Enzyme Commission numbers were assigned to 4,764, 4,509 and 1,560 ORFs, respectively. A gene highly homologous to *tet45* was detected in a contig of 236,180 bp.

### Data availability.

This whole-genome shotgun project was deposited at DDBJ/EMBL/GenBank under the accession number PYAP00000000. The version described here is the first version, PYAP01000000. Raw reads are available in the NCBI SRA under accession number SRX4662930.
